# Barriers and facilitators for providing self-care advice in community pharmacies: a qualitative study

**DOI:** 10.1007/s11096-023-01571-3

**Published:** 2023-04-08

**Authors:** Rian Lelie-van der Zande, Ellen S. Koster, Martina Teichert, Marcel L. Bouvy

**Affiliations:** 1 Department of Pharmacoepidemiology and Clinical Pharmacology, Utrecht Institute for Pharmaceutical Sciences, Utrecht University, Utrecht, The Netherlands; 2 Department of Public Health and Primary Care, Leiden University Medical Center, Leiden, The Netherlands

**Keywords:** Community pharmacy services, Nonprescription drugs, Pharmacist, Pharmacy technicians, Primary health care, Self care

## Abstract

**Background:**

Community pharmacies are easily accessible for self-care advice. Guidelines for providing self-care advice were introduced in several countries, including the Netherlands in the 1990s. Previous studies have indicated room for improvement in self-care advice in daily pharmacy practice.

**Aim:**

To identify barriers and facilitators for providing self-care advice.

**Method:**

Semi-structured interviews were conducted face-to-face or online with pharmacists and pharmacy assistants using a topic guide based on the Theoretical Domains Framework. The interviews were audio-recorded and transcribed verbatim. The transcripts were deductively analysed to identify barriers and facilitators for self-care counselling. COREQ guidelines were followed.

**Results:**

In total, 13 pharmacists and 12 pharmacy assistants were interviewed to reach data saturation. In general, most themes addressed by pharmacists and pharmacy assistants belonged to similar domains. The following domains were frequently mentioned: environmental context and resources (e.g. priority for prescription drugs, privacy, collaboration with general practitioners, access to patients’ records), intentions (providing reliable advice), skills (communication, decision-making), knowledge (ready guideline knowledge), beliefs about consequences (patient safety), social influences (patient awareness of pharmacist role), reinforcement (lack of reimbursement for relatively time-consuming advice).

**Conclusion:**

This study identifies barriers and facilitators for evidence-based self-care advice. Pharmacists should first support pharmacy assistants by helping them keep their knowledge and skills up to date and creating suitable pharmacy preconditions to facilitate improvements in self-care counselling. Second, collaboration with general practitioners regarding minor ailments should be improved.

**Supplementary Information:**

The online version contains supplementary material available at 10.1007/s11096-023-01571-3.

## Impact statements

- Pharmacists should arrange structured training and assessment of knowledge and skills for providing self-care advice for themselves and their team.
- Pharmacists should evaluate the provision of self-care advice in the pharmacy and reciprocal client referrals with general practitioners.
- Pharmacists should raise awareness of the appropriate use of self-care products with their clients.

## Introduction

The World Health Organization defines self-care as the ability of individuals, families and communities to promote health, prevent disease, maintain health and cope with illness and disability, with or without the support of a healthcare provider [1]. Regarding self-care for minor ailments (e.g. allergic rhinitis, heartburn), community pharmacists (CPs) can play a vital role in supporting individuals with health-informed decision-making, as they are among the most accessible healthcare professionals [2].

Since the 1990s, governments have promoted enhancing self-care with over-the-counter (OTC) medicines to transfer some healthcare costs to consumers [3]. Currently, self-care is increasingly regarded as an option to decrease the burden on healthcare providers since, depending on the definition of minor complaints, 5–20% of general practitioner (GP) consultations relate to minor ailments [4–6].

CPs can provide clients (consumers and patients) with evidence-based self-care advice to support their self-care decision-making. In the 1990s, the Dutch professional pharmacists’ organisation introduced evidence-based self-care guidelines with lifestyle and medication advice to standardise self-care advice for minor ailments [7]. In total, 23 guidelines were developed with a protocol for community pharmacy teams to pose relevant questions to the user (*who*), regarding the type of complaint (*what*), duration of symptoms (*how long*), earlier actions taken (*actions*), and medication use (*medication*), introduced as a WWHAM mnemonic.

Pharmacy assistants (PAs) in the Netherlands complete a three-year vocational programme and assist the pharmacist by advising clients on OTC use for minor ailments [8]. Pharmacists complete a six-year MSc at university, followed by a two-year programme in pharmacy practice. CPs are responsible for all care provided in the pharmacy, and PAs work under their supervision. In daily practice, however, the PA primarily delivers self-care advice. CPs and PAs are trained in self-care counselling based on self-care guidelines during their education. Nevertheless, publications have indicated room for improvement in self-care advice in Dutch pharmacies, such as asking WWHAM questions and providing lifestyle advice [9, 10]. Although the results of a simulated patient study on a condition-based self-care question for allergic rhinitis were satisfactory when advice was sought, the results were mediocre for symptom-based questions [11].

To understand which behavioural changes should be targeted to improve self-care counselling, CPs’ and PAs’ perspectives and experiences should be assessed. The Theoretical Domains Framework (TDF) was developed to assess behaviour determinants that should be addressed to achieve change. Its latest version consists of 14 constructs addressing cognitive, affective (emotion), environmental and social influences on behaviour [12, 13]. Thus, this study adopts an explorative, qualitative approach based on the TDF [12, 14].

### Aim

This study aims to identify the barriers and facilitators for providing self-care advice to understand how pharmacist behaviour in self-care counselling can be improved.

### Ethics approval

This study was approved by the Institutional Review Board of the Division of Pharmacoepidemiology and Clinical Pharmacology, Utrecht University (UPF1901, 9 September 2020).

## Method

### Study design and procedure

In this qualitative study, semi-structured interviews were conducted with CPs and PAs in community pharmacies to explore their attitudes towards the pharmacy’s role in self-care counselling. TDF domains were used for topic guide development, coding and analysis [12, 15], and COREQ guidelines were followed.

### Topic guide development

Two topic guides were developed for the semi-structured interviews, one for CPs and one for PAs, based on TDF development studies [12], previous TDF studies conducted in pharmacies [16–18] and the first author’s personal experience of self-care guideline implementation [19, 20]. The TDF includes domains that provide a view of cognitive, affective, social and environmental influences on human behaviour [12]. This study used the second version of the TDF with 14 domains [12]. The TDF domains were used as the main codes (for domain description and application in this study, see Online Resource 4).

The topic guides consisted of open-ended questions for each of the 14 theoretical domains with several prompts for in-depth follow-up questions (Online Resources 1 and 2). The topic guides began with an introduction to the study and concluded with demographic questions. Questions concerning relationships with GPs and reimbursement were discussed exclusively with CPs. Interviews with three CPs and three PAs were used as a pretest.

### Setting

Dutch law regarding the medical treatment agreement (WGBO) regards CPs as healthcare providers with an autonomous duty to correctly serve and inform their patients about medication use according to current guidelines. CPs can delegate self-care advice to PAs, who are usually clients’ first contact at the counter. In 2021, 30% of 2000 Dutch pharmacies were owned by a pharmacy chain [21]. Four out of ten pharmacies employed an additional pharmacist next to the pharmacist-owner or managing pharmacist [22]. On average, the equivalent of 8.4 full-time PAs was employed per pharmacy [23].

### Study participants

Purposive sampling was initially applied to identify CPs with significant interest in self-care based on publications in the pharmacists’ professional journal. When willing to participate by telephone, each CP was asked to invite one of their PAs for a separate interview. This initial contact was followed by snowball sampling, aiming for diversity in location, type of pharmacy and pharmacists’ working experiences. Of the 17 CPs approached, 13 (77%) agreed to participate, and appointments were scheduled to interview the CPs and PAs. The CPs could choose between face-to-face or online video interviews. Interviews were conducted in the Dutch language. Ten interviews were initially planned per profession, followed by additional interviews until data saturation was reached (no new information or insights were gained) [24]. Data saturation was discussed within the research team after 24 interviews.

### Data collection

Interviews were initially conducted in July and August 2020. However, interviewing was interrupted by the COVID-19 pandemic and resumed from November 2021 to January 2022. Six interviews occurred face-to-face at community pharmacies. The remaining interviews were conducted online using a secure online meeting tool, Jitsi. All the interviews were conducted by RL (interview experience) and audio-recorded with informed consent from the participants. The interviews were transcribed verbatim, coded and entered into NVivo 20 (QSR International, Release 1.5.1).

### Data analysis

A coding book was developed, including the TDF domains and corresponding items. The deductive phase began with reading the transcripts. The transcripts were analysed deductively using NVivo 20 with the TDF domains and subthemes to map key determinants [12]. Domain importance was defined by prominence and variation in views. Domains and subthemes were ranked for both CPs and PAs (Online Resource 3) [12]. Within each domain, content analysis was used to identify facilitators and barriers. The domain content for relevant TDF subthemes was summarised (Online Resource 4). Two researchers (EK and RL) coded three interviews independently using the predefined TDF domain coding book and discussed the results and discrepancies during an initial consensus meeting. Disagreements in coding were discussed, an agreement was reached, and the coding book was adapted. Following this discussion, RL and EK coded the fourth interview independently, and disagreements were again discussed during a second consensus meeting. The coding book was adapted accordingly and discussed with the entire research team. The remaining transcripts were coded by one researcher (RL). Domains and illustrating quotes are presented in the subsequent section.

## Results

In total, 13 CPs and 12 PAs from 13 pharmacies were interviewed, and data saturation was reached. Table 1 presents the CPs’ and PAs’ demographic characteristics and the community pharmacies’ features. The mean interview times were 41 min (33–57 min) for CPs and 27 min (20–36 min) for PAs.

**Table 1:** Characteristics of pharmacies, community pharmacists and pharmacy assistants

Number of pharmacies	13
Type of pharmacy	
Chain	2
Other	11
Location	
Town < 50,000 inhabitants	5
Town 50,000–100,000 inhabitants	4
City > 500,000 inhabitants	4
Number of prescriptions per day	
100–200	2
> 200	11
Number of OTC* products sold per day	
< 10	1
10–50	8
> 50	4
Druggist within a distance of 250 m of the pharmacy	10
Supermarket selling OTC* products within 250 m of pharmacy	9
Number of participating community pharmacists	13
Female	10
Position	
Owner/pharmacist	3
Managing pharmacist	6
Locum pharmacist	4
Years of experience in pharmacy	
< 5 years	4
5–10 years	4
> 10 years	5
Number of participating pharmacy assistants	12
Female	12
Years of experience in pharmacy	
< 5 years	4
5–10 years	3
> 10 years	5

*OTC = over-the-counter

### Self-care advice in pharmacy practice

All CPs acknowledged delegating self-care advice to PAs under their supervision. According to all the participants, the CP’s professional role is to provide self-care advice if needed and to be available for consultations with the PA in complex situations, such as symptoms present for one to two weeks, young children, indistinct symptoms, underlying conditions, interacting medication use and frequent purchases of painkillers or heartburn medication*.*

All the respondents aim to provide clients with appropriate advice with or without OTC medication, so they know what to do. Both CPs and PAs aim to provide high-quality advice by following protocols, providing lifestyle advice, checking medication safety and promoting evidence-based products:

*PA03: ‘It is my role to advise the patient, help as much as possible and refer the patient to the GP if needed. And, that doesn’t mean that they always leave with a product, but that they anyway know what to do’.*

### Barriers and facilitators

Considering less relevant domains per professional group, the CPs and PAs scored comparably for domains (Online Resource 3). Table 2 presents the barriers and facilitators that CPs and PAs experience when providing self-care advice.

**Table 2:** Barriers and facilitators for providing self-care advice

TDF domain	Barrier	Facilitator
*Knowledge*	Lack of ready knowledge of minor ailments Belief that asking WWHAM questions always leads to correct advice according to the guideline Lack of guideline update communication	Availability of self-care guidelines Accessibility to computerised protocols for minor ailments and pharmacy-only products Assigning CPs^a^ or PAs^b^ responsible for updates and product introductions Regular guideline advice updates Team app for communicating updates and new product introductions or weekly/monthly newsletter Availability of training on self-care guidelines during CP^a^ and PA^b^ education Regular on-the-job training from CPs^a^ (i.e. in work meetings) Availability of web-based training programmes Annual training on seasonal complaints
*Skills*	Lack of skills training Lack of skills to retrieve sufficient information from consumers/patients Information about different cultural backgrounds and attitudes towards pharmacists lacking in PA^b^ and CP^a^ education	Optimal conditions for self-care advising, such as personal development plans and team training Presence of conversational (verbal and nonverbal), processing and analytical skills Roleplaying within the team or with trainees Assessing patient self-care skills by web-based testing and simulated patient visits
*Professional role and identity*	CPs^a^ underestimating the importance and difficulty of provision of self-care advice	Actively offering advice on self-care and lifestyle CPs^a^ creating optimal pharmacy conditions for self-care advice by PAs^b^ and securing task delegation
*Beliefs about capabilities*	Lack of active CP^a^ support for knowledge and skills training	PAs’ intrinsic motivation PAs feeling capable due to available information and support from colleagues and CPs^a^ Awareness of follow-up questions in addition to WWHAM for correct problem analysis and self-care advice Attention to empathic staff attitudes at the counter Realising the limits of one’s knowledge
*Optimism*	Lack of recognition of the added value of self-care advice in pharmacy from policymakers and healthcare insurers	Consumers/patients return to the pharmacy for self-care advice based on earlier advice Increase of recognition of self-care advice and the added value of pharmacy by CPs^a^ communicating about minor ailments and self-care online or in journals
*Beliefs about consequences*	Compromised medication safety by lack of self-care products registration in electronic patient records Less trust from patients and consumers in information based on guidelines than in information from other sources (e.g. from the internet, advertisement campaigns) Less critical points of sale available (asking fewer questions) for consumers to purchase self-care products	Adding self-care products to electronic patient records enables medication safety monitoring (e.g. interactions, contraindications) Appropriate self-care advice may prevent minor ailments from developing into diseases Lifestyle advice may help to prevent or decrease minor ailment symptoms after stopping self-care medication Appreciating the initiative of the consumer/patient in taking the initiative to research products (e.g. on the internet) improves the attitude of consumers/patients towards advice for a better alternative
*Reinforcement*	Self-care product registration in electronic patient records requiring non-reimbursable time Additional workload for self-care advice not covered by margin from generic self-care products Lack of public communication campaign about the added value of self-care advice in pharmacy	CPs^a^ and GPs^c^ working together to measure the added value of self-care advice in the pharmacy by showing decreasing GP^c^ consultations and healthcare costs
*Intentions*	Time pressure by prioritising prescription medicines and many patients in the waiting area, leading to assistants paying less attention to problem analysis questioning and registering self-care medicines in the pharmacy information system	Adjusting problem analysis questioning to the consumers depending upon openness to self-care advice Adding self-care products to electronic patient records; at least NSAIDs for patients with cardiovascular disease, elderly patients and home-care patients Providing (lifestyle) advice according to self-care guidelines Registration of pharmacy-only questionnaires in the pharmacy information system
*Goals*		Providing easy and timely access to self-care advice in pharmacy Striving for high-quality pharmaceutical care Medication monitoring for patients using chronic medications or having potential contraindications to secure medication safety
*Memory, attention and decision processes*	Asking WWHAM questions without incorporating spontaneous information from consumer/patient Lack of support for correct appraisal of answers to questions	Structured advice according to protocols in self-care guidelines Practising self-care cases in the pharmacy with trainees or team practice during work meetings to iterate knowledge and skills Stickers on pharmacy-only self-care products and products that interact with chronic medications
*Environmental context and resources*	Priority for prescription dispensings prohibiting asking out problem analysis questions Priority for prescription dispensings prohibiting self-care product registration in electronic patient records Consumers/patients not open to receiving advice Language problems Patient attitude towards self-care advice, depending on cultural background Consumers/patients with low health literacy level Lack of privacy at the counter Image with GPs^c^ and consumers/patients that products in the pharmacy are more expensive than at the druggist Effort and time needed to build good relationships with GPs^c^ Lack of pharmacotherapeutic meetings with GPs^c^ about minor complaints as main subject	Availability of consultation areas with sufficient privacy (consulting room or privacy counter) Distance between counters and separation between counters to provide more privacy Background music in the waiting area Measuring loudness of assistants’ voices at the counter and listening to consultations at the counter in the waiting area Communicating indirectly about privacy-sensitive issues when privacy is limited Pharmacy team familiar with various languages in areas with patients from different cultural backgrounds Google Translate app or translator by telephone for communicating when language is not spoken in the pharmacy Visual and verbal communication with low-literacy patients Availability of an electronic pharmacy information system to check interactions and contraindications on patient level Organising logistic processes in pharmacy and implementing innovative logistic developments to provide time for self-care advice Agreement with GPs^c^ in pharmacotherapeutic meetings on self-care protocols in the pharmacy and reciprocal referral policy Agreement with GPs^c^ on registering self-care medicines in patient medication records: specifically which products and patients Product prices discussed with GPs^c^ in pharmacotherapeutic meetings, comparing generic and brand products Offering consumers/patients a choice of more and less expensive products with information about effect and prices
*Social influences*	Lack of healthcare provider profile of CPs^a^ for consumers/patients	Team members supporting one another in providing self-care advice CP^a^ available for advice on complex situations Spontaneous positive feedback from patients Positive feedback from GPs^c^ after referral
*Emotion*	Disappointment when consumers are not open to lifestyle advice	Satisfied customers following up on advice, thus providing positive feelings
*Behavioural regulation*	PAs^b^ not accepting responsibility when receiving feedback	Discussing project results (e.g. pharmacy-only product registration) or knowledge/skill test results, including assessing opportunities for improvement CPs^a^ providing feedback to PAs immediately after incorrect self-care advice CPs^a^ encouraging PAs^b^ to share new information with colleagues CP^a^ encouraging new PAs to listen to self-care advice provided by experienced PAs

^a^*CP* = community pharmacist

^b^*PA* = pharmacy assistant

^c^*GP* = general practitioner

#### Professional role

Several CPs mentioned creating optimal conditions for self-care advice (e.g. knowledge and skills development and pharmacy facilities), securing task delegation, providing feedback to PAs and discussing the importance of appropriate self-care advice with team members:

*CP03: ‘Ensuring that your team has enough expertise and skills, so you have to impose requirements on the training programme: partly team training, partly personal training. Facilities and product assortment should be state-of-the-art’.*

*CP01: ‘A pharmacist should monitor and correct PAs’ advice where needed. When pharmacy assistants start working in the pharmacy, their knowledge and skills levels are comparable, but after six months, the influence of the pharmacist shows; but if pharmacists let it slip, then, yes, of course, quality decreases’.*

#### Intentions

Most PAs mentioned that they do their best to provide clients with reliable advice. However*,* it is the clients’ responsibility if they do not appreciate the advice:

*CP06: ‘They don’t have time nor feel like answering our questions; they think that pharmacy is always asking questions. They prefer to get it at the druggist or supermarket’.*

#### Knowledge

Both CPs and PAs emphasised the importance of the availability and adherence to national evidence-based self-care guidelines for correct self-care advice. A barrier is that most PAs are unaware of guideline medication updates because of a lack of attention in pharmacies. Several CPs assign a team member to track guideline updates and new product introductions or prefer updates in a central electronic decision-support system. Most participants consider their ready knowledge to be reasonable. However, several CPs mentioned as a barrier that PAs’ and CPs’ ready knowledge is insufficient to provide the correct advice for all self-care requests:

*CP01: ‘We should pay more attention to ready guideline knowledge at the counter’.*

In contrast, most PAs are unconcerned about the lack of thorough guideline knowledge because they believe they know where to find information when needed.

All participants responded that they apply the WWHAM protocol for problem analysis. To achieve more straightforward advice, several pharmacies use an electronic decision-support system for asking WWHAM questions since the system also considers lifestyle advice and provides first- and second-choice medications according to the guideline. Most CPs and all PAs believe that the correct advice will be provided when WWHAM questions are asked for condition-based, symptom-based and product-based requests. However, CPs who participated in a simulated patient programme mentioned a barrier: WWHAM questions are not always sufficient for symptom-based requests:

*CP10: ‘No, I don’t think that WWHAM is always sufficient. We learned that from a simulated patient visit on a symptom-based request in our pharmacy. We did not grasp the catch’.*

#### Skills

Next to knowledge of the guidelines, PAs and CPs mentioned the following essential skills for appropriate advice: (1) verbal and non-verbal conversation skills, such as maintaining eye contact, retrieving sufficient information from clients, asking open-ended questions and shared decision-making; (2) processing skills, such as explaining when using a tool or consulting a colleague, deviating from the strict WWHAM order by integrating spontaneous information provided by the client and advising clients to return when they have questions; and (3) analytical skills, such as the ability to interpret answers and estimate when and how long to ask follow-up questions:

*CP04:‘Knowledge, of course, relating to the content but also to products, and communication skills, of course, that you are able to ask open-ended questions and follow up on them and the ability to convey it well’.*

*PA03: ‘You have to listen carefully and listen between the lines because they provide a lot of information spontaneously’.*

*PA03: ‘You always keep eye contact, and you explain to the client what you are doing’.*

*CP10: ‘We trained to customise our advice when implementing the Consultation guideline, trying to attune to what people need, and we found it quite hard’.*

The PAs also mentioned that during education, the emphasis was on communication skills instead of ready knowledge. CPs who implemented knowledge and skills development and assessment predominantly facilitated it by organising web-based training and testing, simulated patient visits, on-the-job training, discussing assessment results in work meetings, assigning accountable PAs and CPs and communicating guideline updates and product introductions. Several CPs and PAs reported roleplaying between the pharmacy team and interns:

*PA10: ‘We also practice when we discuss a minor ailment during work meetings. We always see to it that we discuss that and update our advice accordingly’.*

#### Beliefs about capabilities

The PAs believe that colleagues’ ability to provide correct advice is not defined by years of experience in the pharmacy but depends on intrinsic motivation to maintain and improve knowledge and skills while working. The CPs feel that the extent of the advice could depend on the PA at the counter.

#### Social influences and beliefs about consequences

The PAs observed that clients are unaware of the CP’s healthcare provision role and sometimes have less confidence in evidence-based information from the pharmacy compared to advertisements and the internet. The PAs believe this could lead to suboptimal minor ailment treatment. The PAs also noticed that clients who do not appreciate being asked questions switch to other OTC points of sale, such as druggists and supermarkets, where fewer questions are asked, which could also lead to suboptimal minor ailment treatment.

At the counter, the PAs reported asking their colleagues or the CP for advice when their knowledge was insufficient. Only a few PAs search for information online, while others consider this approach unprofessional. The CPs and PAs mentioned that they learned about minor ailments from guidelines provided during their pre-graduate training, although all reported they learn most in pharmacy practice by listening to experienced PAs.

Several CPs believe the pharmacy team could help patients distinguish between reliable and unreliable information. However, the PAs consider it essential to appreciate clients’ efforts to search for information before discussing an evidence-based alternative. The study participants consider that good advice, with or without self-medication in the pharmacy, could prevent the development of chronic disease and thereby save costs for society. They also believe that access to electronic patient records and the ability to monitor interactions with chronic medications and contraindications facilitates medication safety and provides significant added value for the pharmacy.

#### Reinforcement

The CPs consider time spent providing self-care advice and checking medication safety that is not reimbursed or covered by a margin for generic self-care medication to be a barrier. The CPs also experience the lack of recognition for the added value of self-care in the pharmacy by GPs, clients and healthcare insurers as a barrier.

#### Environmental context

Environmental barriers, such as time pressure caused by a crowded waiting area and prioritising dispensing prescribed medicines, are reasons for omitting WWHAM questions. According to a CP, innovative logistic interventions, such as central filling and a dispensing robot, could free time for self-care advice and on-the-job training. A lack of privacy at the counter is a barrier to PAs retrieving sufficient client information. Study participants observed different client perceptions regarding privacy in the waiting area. In some pharmacies, clients appreciate being offered a consulting room, whereas, in other pharmacies, clients feel embarrassed about visiting it. Several pharmacies have introduced background music in the waiting area, which could affect PAs’ concentration when advising. Furthermore, PAs experiencing the loudness of their voice by sound measurement and listening to consultations at the counter in the waiting area were mentioned as facilitators. Respondents also mentioned language problems and clients with different cultural backgrounds having more confidence in a GP from their country of origin as a barrier. Team members speaking various second languages, using Google Translate and interpreting by telephone were identified as measures to overcome language problems.

According to most CPs, during pharmacotherapeutic meetings with GPs, self-care medication is only discussed when OTC medicines are relevant for treating chronic conditions (e.g. vitamin D or NSAIDs). Clients and GPs perceive pharmacies as more expensive than other OTC points of sale, such as druggists and supermarkets. GPs with whom the CPs discuss self-care in the pharmacy realise that generic OTC products, which pharmacies predominantly advise, are less expensive than branded products. One pharmacist agreed with GPs on a local formulary based on national self-care guidelines. However, most pharmacists doubt whether GPs are aware of pharmacy self-care advice:

*CP06: ‘I never discussed self-care with the GPs, and they may very well not know what we do, but I hope they have any idea of our pharmacy providing self-care advice’.*

## Discussion

### Statement of key findings

The following domains were frequently mentioned as either barriers or facilitators for self-care advice: environmental context and resources (e.g. priority to dispensing prescription drugs by pharmacy staff, privacy, collaboration with GPs and access to patients’ records), intentions (providing reliable advice), skills (communication, decision-making), knowledge (ready guideline knowledge), beliefs about consequences (patient safety), social influences (patient awareness of pharmacist role) and reinforcement (lack of reimbursement for relatively time-consuming advice). In general, the CPs’ and PAs’ perceptions of barriers and facilitators align.

### Study strengths and weaknesses

A significant strength of this study is that it explores CP and PA perspectives. The number of interviews is sufficient to reach data saturation [13]. Although the groups are not representative, sufficient CPs and PAs from various backgrounds (e.g. pharmacy type, location, pharmacist position and years of experience) were interviewed to allow for different local situations and diversity in the responses. In general, the CPs’ and PAs’ perceptions of barriers and facilitators align, adding to data saturation.

The CPs could choose between face-to-face or online video interviews. This choice was supported by a study concluding that completing web-based interviews is a viable alternative to face-to-face interviews [25]. Since a qualitative study aims to collect as many perspectives and experiences as possible, and interviewees can elaborate differently online and face-to-face, two different interview methods could be advantageous. However, comparative perspectives and experiences were expressed in both the online and face-to-face interviews.

The most significant study weakness is that the topic guides were not piloted with CPs and PAs. The first six interviews were considered pilot interviews. Since these interviews only led to changes in the order of the opening questions, they were also included in the analysis.

### Interpretation

It is the CP’s responsibility to ensure that PAs are competent self-care advisers. This competence requires ready knowledge of minor ailments and accurate appraisal of client answers, in which the pharmacy support workforce should be trained. This finding aligns with an earlier study recommending a structured training approach for the entire community pharmacy team to promote the delivery of robust, high-quality minor ailment services [26]. Community pharmacists should provide regular on-the-job training and establish personal development plans for pharmacy assistants to develop their knowledge and skills. Skills and knowledge can be assessed as part of an individual online training or assessment programme or during simulated patient visits [27]. Pharmacy staff self-assess their behaviour with a poor to moderate level of reliability [28]. Teams should discuss assessment results in work meetings to learn from assessments and make arrangements to improve advising [29, 30]. Furthermore, practising roleplay can improve pharmacy assistants’ knowledge and conversational, processing and analytical skills [31, 32].

This study found that CPs rely on the WWHAM mnemonic for problem analysis. Although mnemonics can allow for standardising information obtained from the patient, interpreting the acquired data can be problematic [33]. This difficulty was demonstrated in a simulated patient study on self-care advice for allergic rhinitis symptoms conducted in the Netherlands [11]. Problem analysis requires follow-up questions, correct problem identification and adequate problem-solving [11, 34]. This study’s results align with earlier research findings that CPs exhibit poor clinical reasoning due to reliance on protocol-driven questioning [35–37]. Pharmacy assistants may be inclined to skip WWHAM questions when clients indicate (e.g. by verbal or non-verbal communication) that they do not need additional information. Nevertheless, after explaining the importance of patient safety, pharmacy assistants should at least ask about the person for whom the product is intended and check whether its use is known and the dosage is correct [38].

According to the study participants, registering self-care products in electronic patient medication records enables clinical risk management (e.g. by generating alerts for patients with concomitant morbidity or reduced renal function). As identified in an earlier study, CPs tend to place medication safety first [39]. Their primary motivation is the health and well-being of their patients rather than financial incentives [40]. Earlier research has demonstrated that safety and effectiveness are significant factors for clients when purchasing OTC products [41]. Nevertheless, clients may not be inclined to visit the pharmacy for self-care advice because they are uncertain about the role of community pharmacists [42]. Clients are usually confident about their skills and knowledge of non-prescription medicines [43, 44]. Therefore, community pharmacists should raise awareness among clients about self-care advice in the pharmacy by providing them with appropriate, effective and safe advice to treat minor ailments and behavioural advice to prevent a return of symptoms after stopping non-prescription medicines [17]. Moreover, informing clients about the background of safety-related questions in the pharmacy and the reasons for recording OTC products in the patient record could improve the perception of added value regarding self-care advice in the pharmacy.

For medication safety, community pharmacists add dispensing self-care products to patient records and check for contraindications and for interactions with chronic medication. However, these activities are not currently remunerated in the Netherlands. Thus, preventive behavioural advice and medication safety currently depend upon the community pharmacist’s intrinsic motivation [45]. This situation could explain why time pressure can lead to prioritising prescription medication services over self-care advice.

Collaboration between community pharmacists and GPs is likely influenced by GPs’ attitudes towards patient empowerment, work pressure and, specifically, the workload due to minor ailment consultations and existing interprofessional relationships [46]. Other causes of GP reticence could be fear of missing alarming symptoms or seeing uncomplicated consultations shift to the pharmacy with only challenging and time-consuming consultations remaining. To improve collaboration between GPs and community pharmacists, learning about and understanding each other’s work is essential, including awareness of differences and synergy, reflecting on GPs’ and community pharmacists’ perspectives and creating procedures and routines for clarifying tasks and responsibilities [47]. Therefore, community pharmacists should discuss GPs’ and community pharmacists’ perspectives on self-care, make joint agreements and facilitate client referrals from GP practices to the pharmacy and vice versa.

The study participants observed different client perceptions regarding privacy in the waiting area and visiting a consultation room. In self-care, a perceived lack of privacy could be a barrier to clients sharing information about minor ailment symptoms with CPs [48, 49]. Sufficiently private areas for pharmacy discussions at the counter could be a solution to address varying views on privacy in the pharmacy [48]. Moreover, preventing sound reflection using sound-absorbing materials could improve privacy conditions in pharmacies.

### Further research

Interventions to improve self-care advice should be designed based on the barriers and facilitators identified in this study, and implementation in practice should be studied.

## Conclusion

This study has identified barriers and facilitators for evidence-based self-care advice. Pharmacists should first support pharmacy assistants by keeping their knowledge and skills up to date and creating appropriate preconditions in the pharmacy since both factors are crucial facilitators for effective self-care counselling. Secondly, collaboration with GPs regarding minor ailments should be improved.

## Supplementary Information

Below is the link to the electronic supplementary material.

Supplementary file1 (PDF 147 kb)

Supplementary file2 (PDF 147 kb)

Supplementary file3 (PDF 138 kb)

Supplementary file4 (PDF 132 kb)
