# Supplementary material for: Barriers and facilitators for providing self-care advice in community pharmacies: a qualitative study
Source: Int J Clin Pharm. 2023 Apr 8;45(3):758–68. doi: 10.1007/s11096-023-01571-3 (PMC10082626; doi:10.1007/s11096-023-01571-3)
Supplement: Supplementary file 2 — Supplementary file2 (PDF 147 kb) [file 11096_2023_1571_MOESM2_ESM.pdf]

Online resource 2: Topic List Pharmacy Assistants

Barriers and Facilitators for Evidence-Based Self-Care Counselling in Community Pharmacy

International Journal of Clinical Pharmacy

Rian Lelie- van der Zande, Ellen Koster, Martina Teichert, Marcel Bouvy

Department of Pharmacoepidemiology and Clinical Pharmacology, Utrecht Institute for Pharmaceutical Sciences, Utrecht University, Utrecht, the Netherlands

a.c.a.lelie-vanderzande@uu.nl

Questions	Prompts
**What do you understand by the term 'self-care'?**	
**What do you consider to be the professional role of the pharmacy assistant in self-care according to the self-care guidelines?**<br>***(Professional role, Environmental context, Knowledge, Skills))***	Professional role of the pharmacist in self-care advice according to the self-care guidelines<br>Attitude of assistant towards self-care advice from druggists.<br>Difference between self-care advice from pharmacy and from druggist<br>Referral to GPs according to the self-care guidelines
**What do you consider to be the importance of self-care advice according to the self-care guidelines compared to pharmaceutical care with regard to prescription medicines (e.g. dispensing process, medication review)?**<br>***(Professional role, Environmental context, Social influences, Goals, Intentions)***	Who delivers self-care advice according to the self-care guidelines<br>at the counter<br>Priority of self-care advice according to the self-care guidelines
**How is the self-care process organised in pharmacy?**<br>***(Professional role, Intentions, Knowledge, Skills, Memory, attention and decision processes, Behavioural regulation)***	Task allocation
**What kind of self-care requests do consumers and patients make in your pharmacy (e.g. product-, condition- or symptom-based)?**<br>***(Beliefs about capabilities, Skills, Knowledge, Beliefs about consequences, Intentions, Social influences, Professional role)***	Team agreement on how to handle these questions<br>Types of consumers visiting pharmacy for self-care advice<br>Self-confidence
**What barriers do you experience when advising on self-care according to the self-care guidelines?**<br>***(Knowledge, Skills, Beliefs about capabilities, Intentions, Beliefs about consequences, Environmental context and resources)***	Hurried customers<br>Privacy sensitive minor ailment<br>Other customers listening in<br>Intense work pressure<br>Staff shortages<br>Training

	Dealing with barriers<br>Factors that encourage attention to self-care<br>Use and review of supporting materials
**Self-care guidelines include lifestyle advice. What is your opinion on that?**<br>***(Knowledge, Skills, Professional role, Beliefs about capabilities, Beliefs about consequences, Intentions, Goals, Memory, attention and decision processes, Social influences, Environmental context and resources, Behavioural regulation)***	Dealing with lifestyle advice for minor ailments<br>Team agreements
**What skills do you need to provide self-care advice according to the self-care guidelines?**<br>***(Knowledge, Skills, Professional role, Beliefs about capabilities, Goals, Memory, attention and decision processes, Behavioural regulation)***	Conversation skills<br>Decision making<br>Asking follow-up questions<br><br>Ready knowledge, plus ready knowledge of colleagues<br>Basis of ready knowledge<br>Training during education<br>Updating of knowledge<br>Training of self-care knowledge and skills<br>Assessment of self-care knowledge and skills
**What do you do when experiencing problems providing self-care advice according to the self-care guidelines?**<br>***(Social influences, Professional role, Skills)***	Consultation of colleague, when?<br>Consultation of the pharmacist, when?<br>Agreements within the team about when to consult the pharmacist
**How do you feel after providing a customer with self-care advice according to the self-care guidelines?**<br>***(Optimism, Emotion, Beliefs about consequences)***	Explanation
**Are there any self-care topics that we missed and that you would like to discuss?**	
