# Supplementary material for: Barriers and facilitators for providing self-care advice in community pharmacies: a qualitative study
Source: Int J Clin Pharm. 2023 Apr 8;45(3):758–68. doi: 10.1007/s11096-023-01571-3 (PMC10082626; doi:10.1007/s11096-023-01571-3)
Supplement: Supplementary file 3 — Supplementary file3 (PDF 138 kb) [file 11096_2023_1571_MOESM3_ESM.pdf]

Online resource 3 Mapping of utterances to the Theoretical Domains Framework domains by professional role

Barriers and Facilitators for Evidence-Based Self-Care Counselling in Community Pharmacy

International Journal of Clinical Pharmacy

Rian Lelie- van der Zande, Ellen Koster, Martina Teichert, Marcel Bouvy

Department of Pharmacoepidemiology and Clinical Pharmacology, Utrecht Institute for Pharmaceutical Sciences, Utrecht University, Utrecht, the Netherlands

a.c.a.lelie-vanderzande@uu.nl

Domain#	CPs[1]<br>*n* = 13##	PAs[2]<br>*n* = 12##
**Environmental context and Resources**	156 (= 1)	165 (= 1)
**Professional role and identity***	151 (= 2)	91 (= 2)
**Intentions**	66 (= 3)	23 (= 7)
**Skills**	56 (= 4)	51 (= 3)
**Knowledge**	47 (= 5)	50 (= 4)
**Beliefs about capabilities**	45 (= 6)	36 (= 6)
**Reinforcement****	40 (=7)	3 (= 14)
**Beliefs about consequences**	34 (= 8)	16 (= 8)
**Behavioural regulation**	30 (= 9)	14 (= 10)
**Goals**	20 (= 10)	5 (= 12)
**Social influences*****	16 (= 11)	40 (= 5)
**Emotion**	14 (= 12)	16 (= 8)
**Memory, attention and decision processes**	14 (=12)	13 (=11)
**Optimism**	5 (= 14)	4 (= 13)

[1] CP = Community Pharmacist

[2] PA = Pharmacy Assistant

Domains presented in order of pharmacists. Rank was derived using weighted scores which were derived from the number of utterances divided by the number of participants, to ensure that findings across roles were comparable.

n refers to the number of utterances coded to each domain.

* questions about GPs in topic list pharmacists only

** question about reimbursement in topic list pharmacists only

*** topic list pharmacy assistants containing more questions relevant for social influences
