# Supplementary material for: Barriers and facilitators for providing self-care advice in community pharmacies: a qualitative study
Source: Int J Clin Pharm. 2023 Apr 8;45(3):758–68. doi: 10.1007/s11096-023-01571-3 (PMC10082626; doi:10.1007/s11096-023-01571-3)
Supplement: Supplementary file 4 — Supplementary file4 (PDF 132 kb) [file 11096_2023_1571_MOESM4_ESM.pdf]

Online resource 4: Summary of domain content for TDF sub-themes

Barriers and Facilitators for Evidence-Based Self-Care Counselling in Community Pharmacy, a qualitative study

International Journal of Clinical Pharmacy

Rian Lelie- van der Zande, Ellen Koster, Martina Teichert, Marcel Bouvy

Department of Pharmacoepidemiology and Clinical Pharmacology, Utrecht Institute for Pharmaceutical Sciences, Utrecht University, Utrecht, the Netherlands

a.c.a.lelie-vanderzande@uu.nl

TDF domain	Sub-theme	Summary of domain content
Knowledge<br>(an awareness of the existence of something)	Knowledge	CPs[1]: Knowledge based on national guidelines and implemented in PA[2] and CP[1] education although more attention is paid to communication skills and knowing where to find information than on active knowledge.<br>Most CPs[1] and PAs[2] regard their ready knowledge as reasonable; self-care curriculum during education.<br>PAs[2]: mention knowledge gaps, e.g. seasonal complaints, high number of interactions of OTC[4] medicines and chronic medication, updates of guidelines and new product introductions.
	Procedural knowledge	PAs[2]: use of WWHAM; electronic protocols support shared decision making; if available, generic product advised with explanation that brand products are more expensive.<br>CPs[1] and PAs[2]: WWHAM questions not always sufficient, important to ask supplementary questions.<br>Guideline summaries and Pharmacy Only questionnaires electronically available, limited awareness of updates.
Skills<br>(an ability or proficiency acquired through practice)	Skills	CPs[1] and PAs[2]: verbal and nonverbal communication skills, processing skills such as eye contact when using an electronic device, ability to interprete answers and estimate when and how long to continue asking questions and ability to communicate with patients from different cultural backgrounds or speaking foreign languages e.g in holiday regions were mentioned.
	Skills development	CPs[1]: limited number of pharmacists had a policy on training, online or in pharmacy. Lack of self-care curriculum in CP[1] specialist education.<br>CPs[1] and PAs[2]: Some pharmacies practise roleplay, mainly with trainees.<br>PAs[2]: Trainees learn most in pharmacy if guidance is available, education more theoretic; important to give trainees opportunity to gain experience at the counter under supervision.

	Skills assessment	CPs[1] and PAs[2]: Online programs for assessment and simulated patient visits program available, all not in regular use. When in use also team discussion of results.
Social/ Professional role and identity<br>(a coherent set of behaviours and displayed personal qualities of an individual in a social or work setting)	Group identity	CPs[1]: self-care medication incidentally discussed in pharmacotherapeutic meetings with GPs[3] when relevant for patients with chronic medication, doubts about knowledge of GPs[3] of self-care advice in pharmacy<br>PAs[2]: good image of pharmacy by providing good advice, and doing this at all times.
	Professional boundaries	CPs[1] and PAs[2]: important to know when to refer to GP[3], based on guidelines.
	Professional identity	CPs[1] and PAs[2]: in pharmacy better expertise and medication safety for patients with chronic medication, and contra-indications (e.g. children, pregnancy, breastfeeding).
	Professional role pharmacy assistant	CPs[1]: PAs[2] are an extension of the pharmacist, have more direct contact with consumers/patients and provide self-care advice.<br>PAs[2]: taking care that patients leave the pharmacy contented with a good advice and knowing what to do, referred to GP[3] if needed; adding OTC[4] products to patient record for patients with chronic medication, at least patients with cardiovascular diseases; check of Pharmacy Only medication using a questionnaire.
	Professional role pharmacist	CPs[1]: pharmacists provide self-care advice and should delegate and secure, monitor, and adjust self-care advice, discussing the importance of self-care for pharmacy and creating optimal conditions for knowledge and skills development and facilities in pharmacy.<br>PAs[2]: CP[1] should be available for consultation on complex issues and provision of guideline updates.
Beliefs about capabilities<br>(acceptance of the truth, reality or validity about an ability, talent or facility that a person can put to constructive use)	Professional confidence	CPs[1]: quality of advice depending on which PA[2] advises but confident that correct advice is given when WWHAM is asked according to protocol.
	Self-confidence	CPs[1]: confident they can provide self-care advice, mainly because they regard their ready knowledge as reasonable but if needed they know where to find information;<br>PAs[2]: confident their ready knowledge is reasonable, think they can provide better advice than the druggist, based on personal experience when visiting the druggist. They also know where to find information if necessary.
	Perceived confidence	CPs[1]: some PAs[2] having natural talent for advising, young PAs[2] often having to seek information, older PAs[2] having more expertise.<br>PAs[2]: confidence of colleagues not defined by years of experience but depending upon intrinsic motivation for knowledge development and eagerness to learn.

Optimism<br>(the confidence that things will happen for the best or that desired goals will be attained)	Optimism	CPs[1]: self-care in pharmacy deserves a prominent place in society; measuring and showing added value of self-care advice in cooperation with GPs[3] may increase recognition in GPs[3], consumers, patients and healthcare insurers; belief of users of electronic protocols that this supports PA independent advice.<br>PAs[2]: belief that consumers are provided with correct advice, that asking WWHAM questions will lead to correct advice and that consumers will revisit for other complaints.
Beliefs about consequences<br>(acceptance of the truth, reality, or validity about outcomes of a behaviour in a given situation)	Outcome expectancies	CPs[1]: increase of patient safety by adding self-care medication to patient file; patients can buy self-care medication at other OTC[4] outlets if it's not clear to them why PA asks questions; ample information available on the internet but guidance from pharmacy needed to distinguish between reliable and unreliable information; a minimal advice, e.g. maximum dosage paracetamol or instructions for use, should always be provided; good advice with or without self-medication in pharmacy may prevent development into a chronic disease and high costs for society; self-care advice may decrease GP[3] consultations.<br>PAs[2]: if you keep calm and smile when patients complain, all will turn out fine; important that consumers themselves try to decrease their complaints but sometimes it is better not to wait too long to consult the GP[3] to prevent it from turning into a more serious disease; if patients insist on buying a product that may be not an optimal choice, they can also buy it at the druggist, lifestyle advice may prevent symptoms re-emerging after stopping self-care medication, consumers may trust evidence-based advice from pharmacy less than commercial drug information (e.g. internet, commercials, advertisements), consumers are happy when you take time to advise them; appreciate the initiative of the consumer/patient to research products (e.g. on the internet) to improve the attitude of consumers/patients towards advice for an evidence-based alternative
Reinforcement<br>(increasing the probability of a response by arranging a dependent relationship, or contingency, between the response and a given stimulus)	Incentives, rewards	CPs[1]: good self-care advice leads to a better profile, to customer satisfaction and returning customers, may lead to less GP[3] consultations, can make the profession of PAs[2] more attractive, helps the patient to recover.
	Reinforcement	CPs[1]: self-care advising and checking medication safety take time which is not reimbursed nor covered by margin on generic self-care medication; pharmacists tend to lack commercial interest, most revenues from prescription medicines; GP[3] should be compensated if self-care by GP[3] is transferred to pharmacy (relatively easy GP[3] consult with reimbursement comparable to standard consult).<br>PAs[2]: consumers should know they can consult the pharmacy for minor ailments and the added value of the pharmacy, specifically patients using chronic medication.

Intentions<br>(a conscious decision to perform a behaviour or a resolve to act in a certain way)	Intentions	CPs[1] and PAs[2]: providing high quality advice by using protocols, and providing lifestyle advice and evidence-based products; medication safety improved by adding self-care products to patient file, at least NSAIDs and Pharmacy Only products for elderly and home-care patients; asking about user of product request and advising at least on usage and max dosage; making time for advice even if that leads to working overtime; in case of high workload only most important questions.
Goals<br>(mental representations of outcomes or end states that an individual wants to achieve)	Goal target setting	CPs[1] and PAs[2]: medication safety by checking interactions and contra-indications for elderly patients and adding OTC[4] products and Pharmacy Only self-care medication to patient file; provision of high quality advice; contribution to safe and effective use of self-care products.
Memory, attention and decision processes<br>(the ability to retain information, focus selectively on aspects of the environment and choose between two or more alternatives)	Decision making	CPs[1]: agreement with GPs[3] on guidelines based formulary supports reciprocal referral.<br>CPs[1] and PAs[2]: self-care and Pharmacy Only-protocols available for questioning, advice with or without medication and referral to GP[3], electronic protocols supporting PA[2] in decision making and uniform advice provision.
	Memory attention	CPs[1] and PAs[2]: stickers available on self-care products with potential interactions support use of Pharmacy Only questionnaire and interaction checks in pharmacy information system; practising and discussing self-care cases in pharmacy to repeat knowledge and skills.
Environmental context and resources<br>(any circumstance of a person's situation or environment that discourages or	Environmental stressors	CPs[1] and PAs[2]: work pressuring (e.g. by prioritising dispensing of prescribed medicines, crowded waiting area) can lead to omitting WWHAM questions; consulting room or privacy counter should be offered when required (verbal or nonverbal signs); introduction of background music if possible, screens between counters, measuring of voice volume, PAs listening in the waiting area to colleagues advising at the counter; language may be a problem, specially in areas with low-literacy patients, patients from different cultural backgrounds, e.g. refugee accommodations, or consumers on vacation; hurried consumers may refuse information or not be willing to answer questions; pharmacy image with GPs[3] and patients that products are more expensive in pharmacy than at other OTC[4] outlets.<br>CPs[1]: improvement of logistic processes can decrease number of patients in waiting area and increase time available for advice.

encourages the development of skills and abilities, independence, social competence and adaptive behaviour)		PAs[2]: consumers react both positively and negatively on consultation room offer.
	Organisational culture and climate	CPs[1]: important to create hospitable atmosphere and product display in waiting area to facilitate self-medication; PAs that switch to pharmacy from other professions are mainly trained on location, most of them having a high education level and being very motivated.<br>PAs[2]: pharmacist attaching importance to high level of patient care.
	Person environment interaction	CPs[1]: conversations running smoothly if people are open to it and ask questions; important to discuss what information from internet led a consumer to resolutely ask for a specific product after acknowledging the effort of searching for information; self-care medication incidentally discussed in pharmacotherapeutic meetings with GPs[3] when relevant for chronic conditions, doubts about knowledge of GPs[3] of self-care advice in pharmacy.<br>PAs[2]: patients needing a clear advice, no more than 2 options to choose from; if patients are hurried you should remain calm and ask required questions; if GP[3] refers patient to pharmacy for self-care product, PA should ask what the GP[3] told already; lifestyle advice only working if patient is prepared to listen.
	Resources and material resources	CP[1]s and PAs[2]: use of www.apotheek.nl for patient information, most pharmacies no longer use brochures; access to patients' medication record; PO checklists; self-care guidelines; stickers for Pharmacy Only medicines and for OTC[4] products interacting with chronic medication.
	Target audience	CPs[1] and PAs[2]: mostly young childrens' parents and elderly patients asking advice, people in between mostly asking for a specific product; patients asked about purchase location of OTC[4] medication during medication review consultations.
Social influences (those interpersonal processes that can cause individuals to change their thoughts, feelings, or behaviours)	Social pressure	CPs[1] and PAs[2]: pressure from outside (e.g. commercials, family, friends) that creates unrealistic expectations in consumers requiring to create confidence prior to the advising process; resistance from consumers if they do not understand the background of PA's questions; consumers not aware of role CP[1] as a health care provider
	Social support	CPs[1] and PAs[2]: when PAs[2] are insecure about advice they confer with one another before consulting the pharmacist, mostly in complex situations.

Emotion (a complex reaction pattern, involving experiential, behavioural, and physiological elements, by which the individual attempts to deal with a personally significant matter or event)	Positive/negative affect	CPs[1]: a consumer who is happy with the advice provides the pharmacist with a satisfied feeling; a pharmacist who trained PAs[2] that switched from another job felt proud to offer them a high education level; disappointed feeling when consumers are not open to lifestyle advice.<br>PAs[2]: helping people gives a contented and nice feeling, especially when the consumer intends to follow up on advice.
Behavioral regulation (anything aimed at managing or changing objectively observed or measured actions)	Feedback	CPs[1]: discussing results of knowledge and skills assessment important to identify opportunities for improvement; CP[1] should take time to listen to PA[2] advice and provide feedback in case of incorrect advice, either during of immediately after self-care consultation; CP[1] can encourage PAs[2] to share new insights or information, or to encourage trainees and starting PAs[2] to listen to experienced PAs[2] providing self-care advice.<br>PAs[2]: discussing the number of dispensed Pharmacy Only products and the percentage of Pharmacy Only dispensings entered into the pharmacy information system and deciding on follow-up; positive feedback from patients after they followed up on advice stimulates paying attention to self-care advice.

[1] CP = community pharmacist

[2] PA = pharmacy assistant

[3] GP = general practitioner

[4] OTC = Over The Counter
